# Nucleic Acid-Based Lateral Flow Biosensor for *Salmonella* Typhi and *Salmonella* Paratyphi: A Detection in Stool Samples of Suspected Carriers

**DOI:** 10.3390/diagnostics11040700

**Published:** 2021-04-14

**Authors:** Zulkiply Nor Amalina, Muhammad Fazli Khalid, Sjafri Faizul Rahman, Muhamad Nuramin Ahmad, Mohamad Ahmad Najib, Asma Ismail, Ismail Aziah

**Affiliations:** Institute for Research in Molecular Medicine (INFORMM), Universiti Sains Malaysia, Kubang Kerian 16150, Kelantan, Malaysia; noramalinazulkiply@gmail.com (Z.N.A.); fazlikhalid@usm.my (M.F.K.); faizulsjafri@gmail.com (S.F.R.); nuramin@usm.my (M.N.A.); najib@student.usm.my (M.A.N.); asmainformm@yahoo.com (A.I.)

**Keywords:** multiplex PCR-lateral flow biosensor, point-of-care testing, *Salmonella* Typhi, *Salmonella* Paratyphi A, food handlers, typhoid carriers

## Abstract

A multiplex rapid detection system, based on a PCR-lateral flow biosensor (mPCR-LFB) was developed to identify *Salmonella* Typhi and *Salmonella* Paratyphi A from suspected carriers. The lower detection limit for *S*. Typhi and *S*. Paratyphi A was 0.16 and 0.08 ng DNA equivalent to 10 and 10^2^ CFU/mL, respectively. Lateral flow biosensor was used for visual detection of mPCR amplicons (stgA, SPAint, ompC, internal amplification control) by labeling forward primers with fluorescein-isothiocyanate (FITC), Texas Red, dinitrophenol (DNP) and digoxigenin (DIG) and reverse primers with biotin. Binding of streptavidin-colloidal gold conjugate with the amplicons resulted in formation of a red color dots on the strip after 15–20 min of sample exposure. The nucleic acid lateral flow analysis of the mPCR-LFB was better in sensitivity and more rapid than the conventional agarose gel electrophoresis. Moreover, the mPCR-LFB showed 100% sensitivity and specificity when evaluated with stools spiked with 100 isolates of *Salmonella* genus and other bacteria. A prospective cohort study on stool samples of 1176 food handlers in outbreak areas (suspected carriers) resulted in 23 (2%) positive for *S*. Typhi. The developed assay has potential to be used for rapid detection of typhoid carriers in surveillance program.

## 1. Introduction

Enteric fever is a public health problem in many developing and underdeveloped countries. However, typhoid fever has become relatively rare in developed countries [1]. The majority of cases are caused by *Salmonella* Typhi, followed by *Salmonella* Paratyphi A. The disease is transmitted via consumption of food and water contaminated by individuals who are carriers of the bacteria [2]. According to the World Health Organization (WHO), a carrier is defined as a person who has fully recovered but continues the fecal excretion of *S*. Typhi intermittently for up to a year thereafter [3]. Approximately 1 to 5% of typhoid patients have been confirmed to be carriers when their stools were tested one-year post-infection [3]. They showed no clinical symptoms but have the potential to cause outbreaks in communities. 

Stool culture is the gold standard for diagnosis of typhoid and paratyphoid carriers. However, it takes two to seven days to produce results and requires skilled laboratory personnel to identify the correct bacterial colonies on the selective agar plate [4,5,6,7]. Carriers can also be identified using serological methods such as Vi-ELISA, with a sensitivity and specificity of 95%, compared to 86% by the stool culture method [8]. However, Vi-ELISA is prone to produce false-positive results due to the widespread use of Vi-vaccine which leads to an increase in anti-Vi antibody level [9]. Furthermore, the Vi-ELISA is not commercially available.

Polymerase chain reaction PCR technique has been known to be sensitive, specific, and able to detect multiple pathogens in a single reaction tube [5,10]. Typhoid detection by PCR works by amplifying identified region(s) of the bacterial DNA in blood samples of suspected typhoid patients. Several studies on development and utilization of PCR for typhoid detection concentrated more on detecting *S*. Typhi in blood in symptomatic patients [4,11,12,13,14,15,16]. PCR amplicons are commonly detected and visualized using conventional agarose gel electrophoresis and an ultraviolet (UV) transilluminator, but this method is time consuming and laborious and requires special equipment and skilled personnel. PCR is also reported to be an effective method to amplify the bacterial genes in stool samples [17,18].

Another method is real-time PCR, and the turnaround time of real-time PCR was often reported to be shorter compared to conventional PCR. A real-time PCR study using cytolysin A (clyA) gene to detect typhoidal and paratyphoidal *Salmonella* in blood samples showed a sensitivity of 40%, which was similar with blood culture although both methods revealed high specificity of more than 95% [19]. Another experiment using flagellin C (fliC-d) gene showed sensitivity of 91.4% and specificity of 100% [20]. Real-time PCR had been previously used to detect of typhoidal *Salmonella*, non-typhoidal *Salmonella* and other enteric pathogens in stool samples of gastrointestinal patients with high sensitivity and specificity of more than 95% [21,22,23]. However, real-time PCR has the limitation that the available master mix reagents and consumables are provided according to the real-time PCR system.

In terms of lateral flow assay development for diagnostics application, several studies were conducted for antibody detection for screening of typhoid and paratyphoid fever [24,25]. A very sensitive confirmatory test is via PCR. PCR has the advantage of adopting lateral flow assay for visualization for amplicons instead of agarose gel electrophoresis. Therefore, an alternative method for laboratory diagnosis of typhoidal and paratyphoidal *Salmonella* is by performing conventional PCR followed by detection by a lateral flow biosensor (LFB) device; this has comparable turnaround time as real-time PCR since the detection for lateral flow is done in 15–20 min [26]. LFB involves the immobilization of antibody on a solid surface, hybridization of labeled-PCR amplicons and detection by nanoparticles such as colloidal gold [27,28,29,30,31,32]. The detection method is rapid, user-friendly and the results can be observed by naked eye [29,32,33,34,35,36]. 

The present study describes a multiplex PCR-lateral flow biosensor (mPCR-LFB) for the detection of *S*. Typhi and *S*. Paratyphi A using labeled primers. The LFB strip utilized five dots which targeted PCR amplicons of *S*. Typhi, *S*. Paratyphi A, pan-*Salmonella*; an internal amplification control (IAC) and anti-biotin antibody as test control. The analytical sensitivity of the mPCR-LFB was determined at DNA and bacterial levels. Evaluation of this assay was performed on stool samples spiked with *S*. Typhi, *S*. Paratyphi A, other *Salmonella* serovars and other gram-negative bacteria; as well as on stool samples collected from food handlers during outbreaks in Kelantan, Malaysia.

## 2. Materials and Methods 

### 2.1. Reagents and Apparatus

Goat anti-mouse IgG (whole molecule)-biotin and mouse monoclonal anti-FITC were purchased from Sigma Aldrich (St. Louis, MO, USA); anti-digoxigenin was from Roche (Penzberg, Germany); anti-Texas red and anti-dinitrophenyl-KLH were from Invitrogen (Carlsbad, CA, USA), and streptavidin-colloidal gold conjugate (40 nm) was from Kestrel BioSciences (Carlsbad, CA, USA). Other materials used in this study were plastic adhesive backing card from G&L Precision Die-cutting (Amstelveen, Netherlands), Unisart CN95 nitrocellulose membrane from Sartorius (Goettingen, Germany), C048 absorbent pad from Millipore (Burlington, MA, USA), No. 8964 conjugate pad from Ahlostrom (Helsinki, Finland), and No. 319 sample pads from Ahlstrom (Helsinki, Finland). Agarose, deoxynucleotide triphosphates (dNTPs), *Taq* DNA polymerase and a 25 bp DNA ladder were purchased from Promega (Madison, WI, USA). 

All primers used in this study were designed using Primer Explorer V4 software, targeting the specific regions of *Salmonella* Typhi genome retrieved from the GenBank (Table 1). The labeled primers were synthesized by Bioneer Corporation (Daejeon, Korea). The running buffer for the lateral flow biosensor contained 1% bovine serum albumin (BSA) and 0.05% Tween-20 in phosphate buffered saline, pH 7.4 (PBS). The assembled lateral flow biosensor was cut into strips using an automatic strip cutter (Kinematic Automation, Sonora, CA, USA). PCR amplification reactions were performed in a PTC-200 MJ Research Thermal Cycler (Ramsey, MN, USA), and amplicons were analyzed using a Syngene UV transilluminator (Cambridge, UK).

### 2.2. Collection of Bacterial Strains and Stool Samples

A total of 100 bacterial isolates, including 25 *S.* Typhi, 25 *S.* Paratyphi A, 25 other *Salmonella* serovars, and 25 non-*Salmonella*, were stock cultures kept at the Institute for Research in Molecular Medicine (INFORMM), Universiti Sains Malaysia. A total of 1176 stool samples from food handlers and suspected carriers collected from the Kelantan Public Health Laboratory Kota Bharu, Kelantan, were used in the test evaluation. All samples were obtained with informed consent from food handlers and suspected carriers, and ethical clearance was obtained from the Human Ethics Committee, Universiti Sains Malaysia (USMKK/PPP/JEPeM [226.4.(1.9)]) dated 2 June 2010.

### 2.3. Preparation and Extraction of DNA from Stool Samples

Spiked stool samples were prepared by adding 1 g of stool sample and 1 mL of bacterial culture to a final volume of 20 mL in Selenite F broth. Serial dilutions of bacterial cultures were used to determine the analytical sensitivity. A total of one gram of stool sample was collected from each food handler and suspected carrier and cultured in a final volume of 20 mL in Selenite F broth for the enrichment and selection. The samples were further incubated at 37 °C for 24 h. Then, 1 mL of bacterial culture from the upper layer of Selenite F broth was transferred into a new 1.5 mL tube. The DNA was extracted using the boiling method [11].

### 2.4. Multiplex PCR Assay

A multiplex PCR assay for *S*. Typhi and *S*. Paratyphi A in the presence of *Salmonella* genus and a non-competitive internal amplification control (IAC) was carried out in a final volume of 20 μL. The reaction mixture contained 1X colorless GoTaq Flexi buffer; 200 μmol/L dNTPs mix; 3 mM MgCl2; 0.5 μM FITC_stgAF and Biotin_stgAR primers; 0.5 μM Texas red_SPAintF and Biotin_SPAintR primers; 0.3 μM DNP_ompCF and Biotin_ompCR primers; 0.7 μM DIG_HemMP and Biotin_18R-1 primers; 1.5 U of GoTaq DNA Polymerase; and 40 pg of an IAC plasmid containing the hemM gene.

PCR was performed using 1 μL of extracted DNA sample in a thermal cycler with the following cycling parameters: 94 °C for 5 min, followed by 25 cycles of denaturation at 94 °C for 30 s, annealing at 61 °C for 1 min 30 s, and elongation at 72 °C for 1 min, with a final extension at 72 °C for 7 min.

A positive control (containing 10 ng of a DNA template of *S*. Typhi, *S*. Paratyphi A and the IAC) and a negative control (containing only a DNA template of the IAC) were included in the mPCR assay. The amplification products were detected by two methods: (i) 3% agarose gel electrophoresis in the presence of 0.5 μg/mL ethidium bromide visualized under UV transilluminator and photographed using an image analyzer and (ii) a lateral flow biosensor.

### 2.5. Preparation of the Lateral-Flow Biosensor Strip

**Figure 1:**
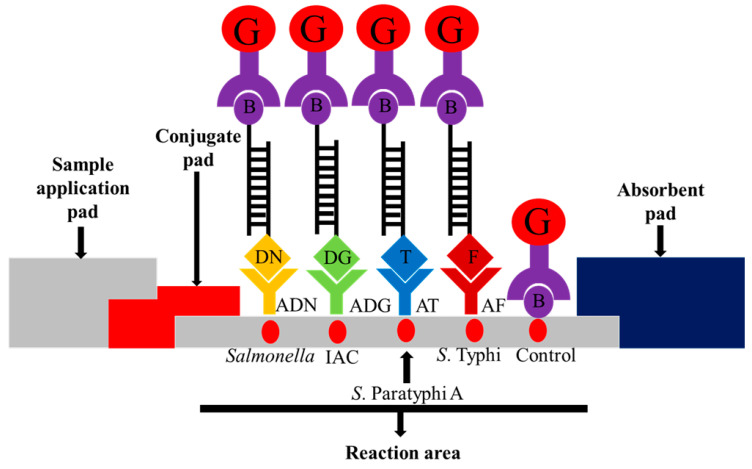
Schematic illustration of the principle of the multiplex PCR-lateral flow biosensor (mPCR-LFB) for the visualization of PCR amplicons using immobilized capture reagents on the nitrocellulose membrane. The target PCR amplicon for pan-*Salmonella* is dinitrophenyl and biotin labeled and is captured on the *Salmonella* reaction area; *S.* Paratyphi A is Texas red and biotin labeled and is captured on the *S.* Paratyphi A reaction area; *S.* Typhi is FITC and biotin labeled and is captured on the *S.* Typhi reaction area, and the internal amplification control (IAC) is digoxigenin and biotin labeled and is captured on the IAC reaction area. The accumulation of streptavidin-colloidal gold conjugate in the respective areas produced visible red dots. Excess streptavidin-colloidal gold conjugate is captured on the control reaction area for the validation of the lateral flow biosensor. G = streptavidin-colloidal gold conjugate; B = biotin; DN = dinitrophenyl; ADN = anti-dinitrophenyl; DG = digoxigenin; ADG = anti-digoxigenin; T = Texas red; AT = anti-Texas red; and F = FITC; AF = anti-FITC.

The lateral flow biosensor strip (5 mm × 73 mm) was assembled by placing a sample pad, conjugate pad, nitrocellulose membrane and absorbent pad on a plastic adhesive backing card to provide a solid support for the strip. The streptavidin-colloidal gold conjugate was placed onto the conjugate pad in the presence of trehalose as a stabilizer. The assembled pads were cut into strips of 5 mm width using an automatic strip cutter. Five capture reagents were dotted onto the nitrocellulose membrane: (i) anti-mouse IgG-biotin antibody (biotin) as control; (ii) mouse monoclonal anti-FITC (anti-FITC) as the *S*. Typhi target; (iii) monoclonal anti-Texas red as the *S*. Paratyphi A target; (iv) anti-dinitrophenyl-KLH, rabbit IgG fraction (anti-DNP) as the pan-*Salmonella* target; and (v) anti-digoxigenin (anti-DIG) as the IAC target. The schematic diagram is shown in Figure 1.

### 2.6. Visual Detection of PCR Amplicons Using the Lateral Flow Biosensor

After PCR amplification, 10 μL PCR product and 150 μL running buffer were added to the sample pad of the biosensor strip. The mixture migrated towards the absorbent pad by capillary action. After 15 min, the result was read based on the presence of red dots on the LFB strip. The presence of five red dots on the positive control strip (strip control, *S*. Typhi, *S.* Paratyphi A, *Salmonella* genus and IAC) and two target dots (strip control and IAC targets) on the negative control strip indicate that the reaction was valid.

### 2.7. Validation of the Primers for the mPCR-LFB

The performance of the primers was validated by calculating the sensitivity and specificity of the mPCR-LFB using genomic DNA extracted from 100 bacterial isolates, including 25 *S*. Typhi, 25 *S*. Paratyphi A, 25 other *Salmonella* serovars and 25 non-*Salmonella* serovars.

### 2.8. Determination of Analytical Sensitivity and Validation of the mPCR-LFB

The analytical sensitivity of the mPCR-LFB was determined at the DNA level using genomic DNA extracted from American Type Culture Collection (ATCC) strains of *S*. Typhi (ATCC 7251) and *S*. Paratyphi A (ATCC 9150). Bacterial counts were also performed to determine CFU/mL. The genomic DNA was serially diluted (two-fold dilutions) with 10 mM Tris-HCl, pH 8.0, at concentrations ranging from 10 ng to 0.02 ng prior to multiplex PCR. The limit of detection (LoD) of the mPCR-LFB was also validated using DNA extracted from stool samples spiked with serially diluted *S*. Typhi and *S*. Paratyphi A culture (10^7^ to 10^1^ CFU/mL). The analytical sensitivity and the LoD for the mPCR-LFB were compared with mPCR-agarose gel electrophoresis (mPCR-AGE). The sensitivity and specificity of the mPCR-LFB were validated using DNA extracted from 100 stool samples spiked with 10^7^ CFU/mL bacterial isolates, including 25 *S*. Typhi, 25 *S*. Paratyphi A, 25 other *Salmonella* serovars and 25 non-*Salmonella* serovars.

### 2.9. Detection of Carriers among Food Handlers Using the mPCR-LFB and Culture Method

The mPCR-LFB was tested with 1176 stool samples of food handlers without history of typhoid and suspected carriers with a history of typhoid. PCR amplicons were detected by both AGE and LFB, and their data were compared. The five capture reagents were immobilized onto the nitrocellulose membrane, and the final format of test is in line-format. The culture method followed by biochemical test was also performed for each sample according to the standard microbiological techniques.

## 3. Results

### 3.1. mPCR-LFB

The biosensor was designed to detect double-stranded DNA sequences that were labeled at both 5′-ends of each strand, one of the labels being biotin and another comprising a dye or DIG. The dyes were (i) FITC for stgA gene of *S*. Typhi, (ii) Texas red for intergenic region of SSPA1723a and SSPA1724 of *S*. Paratyphi A, and (iii) DNP for ompC gene of pan-Salmonella. The latter was included as a control, whereby any amplification of *S*. Typhi/*S*. Paratyphi A must be accompanied by amplification of ompC gene. The DIG was used as capture of IAC amplicon, and its absence indicates false negative results due to incorrect PCR mixture, thermal cycler malfunction, or presence of inhibitors [37]. The amplified PCR products for *S*. Typhi (70 bp), *S*. Paratyphi A (93 bp), Salmonella genus (146 bp), and IAC (123 bp) were observed on agarose gel.

### 3.2. Validation of the Primers for the mPCR-LFB

The mPCR-LFB was tested with genomic DNA extracted from 100 isolates of Salmonella serovars, including *S*. Typhi and *S*. Paratyphi A, and non-Salmonella isolates. All amplicons were detected by both the lateral flow biosensor and agarose gel electrophoresis. The results showed that both the mPCR-LFB and mPCR-AGE were 100% sensitive and specific (Table 2).

### 3.3. Comparison of the Analytical Sensitivity of the mPCR-LFB and mPCR-AGE

**Figure 2:**
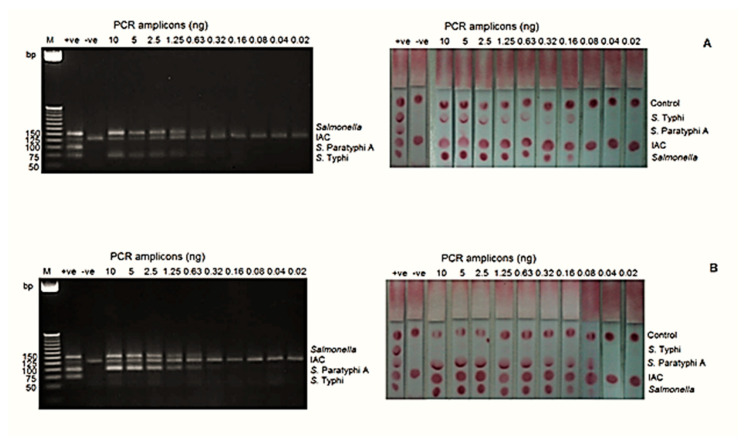
The analytical sensitivity of the mPCR-LFB and mPCR-AGE was determined using different concentrations of PCR amplicons ranging from 0.02 to 10 ng. (**A**) The analytical sensitivity using genomic DNA of *S.* Typhi was 0.63 ng using mPCR-AGE and 0.16 ng using the mPCR-LFB. (**B**) The analytical sensitivity using genomic DNA of *S.* Paratyphi A was 0.63 ng using mPCR-AGE and 0.08 ng using the mPCR-LFB. M= 25 bp markers, +ve= positive control, −ve= negative control.

The analytical sensitivities at the DNA level for *S*. Typhi and *S*. Paratyphi A by mPCR-LFB were 0.16 ng and 0.08 ng, respectively, whereas mPCR-AGE showed that both serovars can be detected as low as 0.63 ng (Figure 2).

### 3.4. Limit of Detection and Evaluation of the mPCR-LFB Using Spiked Stool Samples

**Figure 3:**
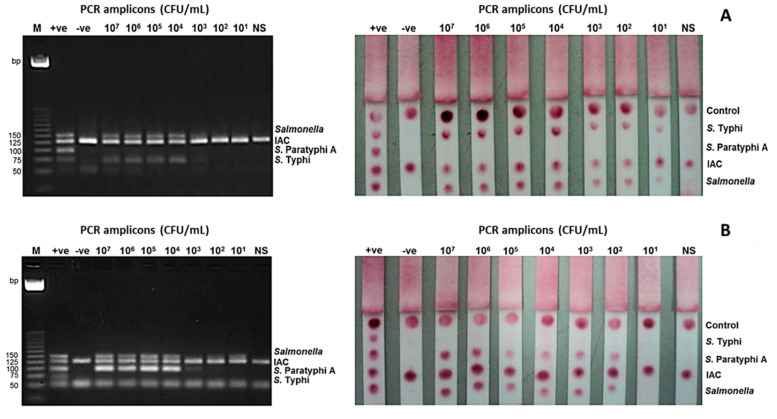
Limit of detection (LoD) of the mPCR-LFB and mPCR-AGE were determined using different concentrations of PCR amplicons ranging from 10^7^ to 10^1^ CFU/mL. (**A**) LoD using DNA of *S.* Typhi was 10^4^ CFU/mL using mPCR-AGE and 10^1^ CFU/mL using mPCR-LFB. (**B**) LoD using DNA of *S.* Paratyphi A was 10^4^ CFU/mL using mPCR-AGE and 10^2^ CFU/mL using mPCR-LFB. M= 25 bp markers, +ve= positive control, -ve= negative control.

The limit of detection (LoD) of the mPCR-LFB at the bacterial level was determined using stool samples spiked with *S*. Typhi and *S*. Paratyphi A. It was found to be 10^1^ CFU/mL for *S*. Typhi and 10^2^ CFU/mL for *S*. Paratyphi A, while the detection limit of mPCR-AGE was 10^4^ CFU/mL for both *S*. Typhi and *S*. Paratyphi A (Figure 3).

Evaluation mPCR-LFB was performed using stool samples spiked with 100 bacterial isolates, including 25 *S*. Typhi, 25 *S*. Paratyphi A, 25 other *Salmonella* serovars and 25 non-*Salmonella* serovars, and the result showed 100% sensitivity and specificity. The mPCR-LFB results were also fully consistent with those of mPCR-AGE.

### 3.5. Performance of Carriers’ Detection among Food Handlers Using the mPCR-LFB Compared to Culture Method

**Figure 4:**
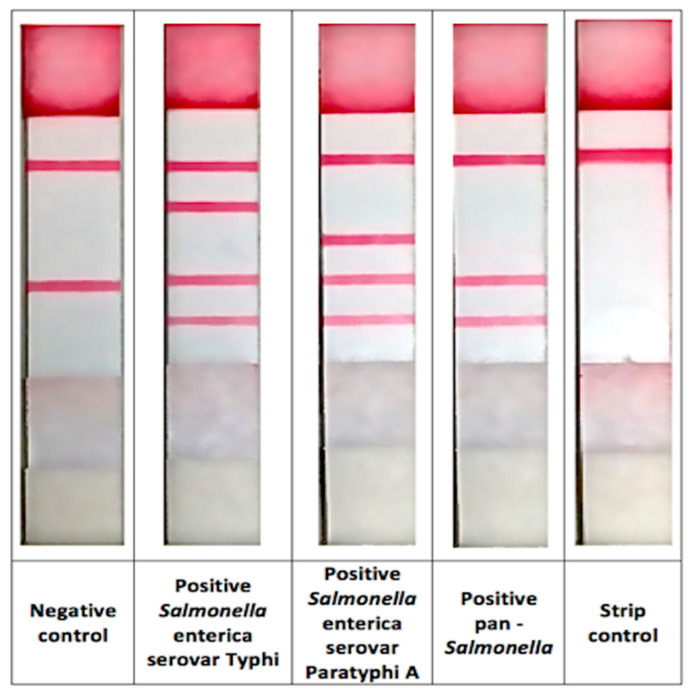
The representative of the mPCR-LFB in line-format.

The mPCR-LFB was applied to detect *S*. Typhi and *S*. Paratyphi A using DNA extracted from 1176 stool samples of food handlers besides the routine culture method. The representative of the mPCR-LFB in line-format is shown in Figure 4. The method was able to detect *S*. Typhi in 23 (2.0%) samples and *S*. Paratyphi A in 3 (0.3%) samples while the culture method detected *S*. Typhi and *S*. Paratyphi A in only 3 (0.3%) and 1 (0.1%) sample, respectively (Table 3). There was no PCR inhibitor detected in all samples.

## 4. Discussion

In this study, PCR-lateral flow biosensor was developed in which PCR product was applied on the sample pad of a lateral flow strip together with running buffer. The mixture rehydrated and released the adjacent dried streptavidin-colloidal gold conjugate, followed by binding to the 5′-biotin labeled amplicons. The complex then moved up the membrane strip by capillary action. The PCR amplicons that were labeled with 5′-FITC, 5′-Texas red, 5′-DNP or 5′-DIG then bound with their respective capture reagents immobilized on the membrane. The accumulation of streptavidin-colloidal gold conjugate at the respective areas was visualized as a red dot for test, while conjugation bound with biotin at the control zone indicated the proper functioning of the lateral flow biosensor [32,38].

Colloidal gold nanoparticles were used in this lateral flow biosensor due to its high affinity and ease to be functionalized on biomolecules. The advantages of gold nanoparticles include good stability, high affinity towards biomolecules, environmentally friendly, and having an intense red color that can be easily detected by the naked eye or by a reader [38,39,40,41,42,43]. The mPCR-LFB detected and indirectly visualized the amplified product of *S*. Typhi or *S*. Paratyphi A by formation of red dots after 15 min of amplicons application onto the lateral flow strip. The biosensor was designed to allow both primers to be labeled instead of using a labeled probe, thus eliminating the need for the DNA strands to be denatured and single stranded DNA hybridized to the labeled probe.

The diagnosis of a chronic carrier using culture of stool sample and confirmation by biochemical test and serology is time consuming as it needs 2–7 days to produce results. Application of PCR after sample enrichment reduces the time taken, and the more recent introduction of lateral flow assay as an alternative to agarose gel electrophoresis further reduces the turnaround time from four hours to two hours [23].

The primers derived from the genes revealed that the primers were highly specific since the test did not cross-react with other bacteria; thus, the assay has potential to be used as a confirmatory test for detection of *S*. Typhi and *S*. Paratyphi A from suspected typhoid carriers. Validation or evaluation with clinical isolates is necessary for prototype development as supported by previous studies that also incorporated *S*. Typhi, *S*. Paratyphi A, other *Salmonella* spp. and other related bacteria to ensure the higher level of sensitivity and specificity before testing with clinical samples [20,44,45].

Detection by the lateral flow biosensor was 4- and 6-fold more sensitive compared to conventional agarose gel electrophoresis for detection of *S*. Typhi and *S*. Paratyphi A, respectively. In terms of the load of bacteria (CFU/mL) spiked in the stool samples, detection by PCR-LFB was found to be in the range of 10 to 10^3^-fold more sensitive compared to PCR-AGE. Therefore, detection of PCR amplicons by lateral flow biosensor proved to be more sensitive than agarose gel electrophoresis. The results are supported by previous studies on other diseases, which showed that the lateral flow biosensor was more sensitive than agarose gel electrophoresis [34,46,47].

The sensitivity and specificity of mPCR-LFB in this study revealed high sensitivity and specificity of 100% when tested with spiked stool samples. Furthermore, the performance of the mPCR-LFB in prospective study in stool samples of food handlers showed a higher percentage of positivity by mPCR-LFB compared to culture method. Higher percentage of positivity in mPCR-LFB compared to culture method (*S* Typhi: 2.0% vs. 0.3%; *S*. Paratyphi A: 0.3% vs. 0.1%) suggests that the test has potential to be used as an alternative tool for detecting the typhoid carriers from stool samples of asymptomatic food handlers or suspected carriers in typhoid outbreak hotspots.

Similar studies had been conducted and have also shown the successful development of nucleic acid-based assays for detection of *Salmonella* spp. and other gastrointestinal bacteria in poultry products and stools of suspected gastrointestinal patients [21,22,48]. The studies demonstrated molecular methods are highly sensitive and specific by utilizing multiplex PCR-agarose gel and multiplex real-time PCR for the detection of the bacteria compared to gold standard culture method which is used as routine microbiological method for isolation and identification of the bacteria in clinical samples. The studies showed sensitivity and specificity of more than 95% suggesting the high accuracy of the developed assays. The above-mentioned studies focused on the nucleic acid assays for non-typhoidal Salmonella and other gastrointestinal bacteria whereas this mPCR-LFB focused on the detection of typhoid carriers.

The other study had utilized multiplex real-time PCR to detect *S.* Typhi and *S.* Paratyphi in stool samples [22] with the analytical sensitivity of 10^3^ CFU/mL for both bacteria. This mPCR-LFB has showed better performance with a sensitivity of 10 and 10^2^ CFU/mL for *S*. Typhi and *S*. Paratyphi, respectively. In addition to that mPCR-LFB also offers benefit over multiplex real-time PCR as require cheaper cost of equipment and lower technical skilled lab technicians to operate and interpret the results.

The finding of this study suggested that mPCR-LFB has the potential to be used as a diagnostic tool for carrier detection. Furthermore, this assay is simple, rapid, highly sensitive, and specific and does not require any tedious procedure or sophisticated equipment.

## 5. Conclusions

The mPCR-LFB developed in this study was able to fulfill most of WHO criteria of a diagnostic test suitable for low resource settings, i.e., rapid, robust, sensitive, specific, and user-friendly. It showed good potential for detecting carriers among food handlers and suspected carriers and thus merits further validation studies.

## Figures and Tables

**Table 1:** Primers used in the study.

Primer	Primer Sequence	5′-Label	Target Gene (Accession Number)	Target Bacterium	Amplicon Size (bp)
FITC_ stgAF	TGATGGCACCGTTCACTTCCTTG	FITC	*stgA* (AL627280.1)	*S.* Typhi	70
Biotin_ stgAR	ATCAGCGGTTTGTGGCGTAAC	Biotin			
Texas red_ SPAintF	CGAACCTGGCAACATACCATTAGAT	Texas red	Intergenic region between SSPA 1723a and SSPA 1724 (FM200053.1)	*S.* Paratyphi A	93
Biotin_ SPAintR	TGCCTCAAATCATCAGTAATCTCTC	Biotin			
DNP_ ompCF	GCAGCGTGAGCGGTGAAAACAC	DNP	*ompC* (NC_006511)	Pan-*Salmonella*	146
Biotin_ ompCR	GTTCTGATCGGCAGTACGTTTAG	Biotin			
DIG_ IACF	GCAGATATTAGGACAAGTTAAGCAAG	DIG	*hemM* (AF22752)	Non-competitive IAC	123
Biotin_ IACR	GTTTCTGTTCTTACCCGTTTC	Biotin			

**Table 2:** Summary of the results of mPCR-LFB and mPCR-agarose gel electrophoresis (mPCR-AGE) using genomic DNA extracted from 100 isolates of different bacteria strains.

Strains (n = 100)	No. of Strains	No. of Positive Test					
		mPCR-LFB Results			mPCR-AGE Results		
		stgA	SPAint	OmpC	stgA	SPAint	OmpC
*Salmonella* Typhi	25	25	0	25	25	0	25
*Salmonella* Paratyphi A	25	0	25	25	0	25	25
**Other *Salmonella* serovars**							
*Salmonella* Branderup	1	0	0	1	0	0	1
*Salmonella* Choleraesuis	1	0	0	1	0	0	1
*Salmonella* Paratyphi B	2	0	0	2	0	0	2
*Salmonella* Paratyphi C	1	0	0	1	0	0	1
*Salmonella* Typhimurium	1	0	0	1	0	0	1
*Salmonella* Walter	1	0	0	1	0	0	1
*Salmonella* Farsta	1	0	0	1	0	0	1
*Salmonella* Richmond	1	0	0	1	0	0	1
*Salmonella* Bordes	1	0	0	1	0	0	1
*Salmonella* Bordeaux	1	0	0	1	0	0	1
*Salmonella* Ayton	1	0	0	1	0	0	1
*Salmonella* Virchow	1	0	0	1	0	0	1
*Salmonella* Rissen	1	0	0	1	0	0	1
*Salmonella* Idikan	1	0	0	1	0	0	1
*Salmonella* Abony	1	0	0	1	0	0	1
*Salmonella* Albert	1	0	0	1	0	0	1
*Salmonella* Eppendorf	1	0	0	1	0	0	1
*Salmonella* Corvallis	1	0	0	1	0	0	1
*Salmonella* Poona	1	0	0	1	0	0	1
*Salmonella* Heidelberg	1	0	0	1	0	0	1
*Salmonella* Emek	1	0	0	1	0	0	1
*Salmonella* Kissi	1	0	0	1	0	0	1
*Salmonella* Djakarta	1	0	0	1	0	0	1
*Salmonella* Bareilly	1	0	0	1	0	0	1
**Other bacterial strains**							
*Acinetobacter baumanii*	1	0	0	0	0	0	0
*Citrobacter freundii*	1	0	0	0	0	0	0
*E. coli*	1	0	0	0	0	0	0
EHEC	1	0	0	0	0	0	0
EIEC	1	0	0	0	0	0	0
EPEC	1	0	0	0	0	0	0
*Klebsiella pneumoniae*	1	0	0	0	0	0	0
*Proteus mirabilis*	1	0	0	0	0	0	0
*Proteus vulgaris*	1	0	0	0	0	0	0
*Pseudomonas aeruginosa*	1	0	0	0	0	0	0
*Shigella boydii*	1	0	0	0	0	0	0
*Shigella dysenteriae*	1	0	0	0	0	0	0
*Shigella flexneri*	1	0	0	0	0	0	0
*Shigella sonnei*	1	0	0	0	0	0	0
*Vibrio cholerae*	3	0	0	0	0	0	0
*Yersinia enterocolotica*	1	0	0	0	0	0	0
TOTAL	100	25	25	75	25	25	75

Sensitivity and specificity: 100%, n = number of strains.

**Table 3:** Summary of mPCR-LFB and culture method for stool samples collected from food handlers and suspected carriers.

N = 1176 (Stool Samples)	*Salmonella* Typhi (Percentage Positivity)	*Salmonella* Paratyphi A
Culture method	3 (0.3%)	1 (0.1%)
mPCR-LFB	23 (2.0%)	3 (0.3%)
